# Short-Term Efficacy of Laparoscopic Treatment for Colorectal Cancer in Patients with Schistosomiasis Japonica

**DOI:** 10.1155/2016/8357025

**Published:** 2016-10-30

**Authors:** Zhu Yi, Jiang Hong-Gang, Chen Zhi-Heng, Lu Bo-Hao

**Affiliations:** Department of Surgical Oncology, First Hospital of Jiaxing, Jiaxing, Zhejiang 314000, China

## Abstract

*Introduction*. Schistosomiasis is associated with numerous complications such as thrombocytopenia, liver cirrhosis, portal hypertension, and colitis. To the best of our knowledge, the feasibility and outcomes of laparoscopic colorectal surgery in patients with schistosomiasis have not yet been studied.* Methods*. In this study, the data of 280 patients with colorectal carcinoma along with schistosomiasis japonica infection who underwent laparoscopic or open colorectal surgery were retrospectively analyzed. Preoperative data, operative data, pathological outcomes, postoperative complications, and recovery were compared between patients in the laparoscopic (LAC) and open (OC) groups.* Results*. There were no significant differences in the preoperative data between the groups. However, fewer postoperative complications, especially severe hypoproteinemia, early postoperative feeding, and shorter postoperative hospital stay, were observed in patients in the LAC group (*P* < 0.001). The mean operative time was higher in the LAC group (180 min versus 158 min; *P* < 0.001), while the mean blood loss was similar (95 mL versus 108 mL; *P* = 0.196) between groups. The mean number of lymph nodes harvested was also similar in both groups (15 versus 16; *P* = 0.133).* Conclusion*. Laparoscopic surgery for colorectal cancer is safe in patients with schistosomiasis japonica and has better short-term outcomes than open surgery.

## 1. Introduction

Schistosomiasis is an endemic communicable disease prevalent in tropical and subtropical areas of Asia and Africa [1]. According to data published by WHO, more than 61.6 million people were reported to have been treated for schistosomiasis in 2014 [2]. Despite the availability of effective treatment for more than 50 years,* Schistosoma japonicum* continues to be a major public health concern in China [3]. Schistosomiasis is associated with numerous complications, including thrombocytopenia, leucopenia, liver cirrhosis, portal hypertension, varicose veins, and splenomegaly [4, 5]. Moreover, it is known to cause colonic disease manifested as acute colitis, chronic colitis, and chronic active colitis and to predispose individuals to the development of colorectal cancer [6, 7]. These factors make the surgical treatment of schistosomiasis more difficult. However, whether this infection affects the feasibility and outcomes of laparoscopic colorectal surgery has not yet been studied.

This study aimed to evaluate the short-term efficacy of laparoscopic surgery for colorectal carcinoma in patients with schistosomiasis.

## 2. Materials and Methods

**Figure 1:**
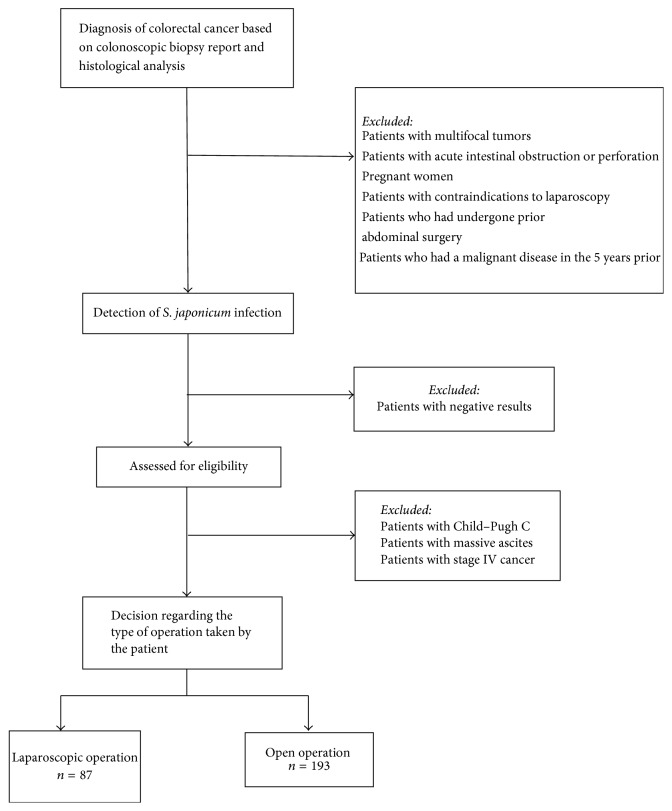
Flowchart of patient selection.

This study is a retrospective review of patients with a history of schistosomiasis japonica infection who underwent surgical resection for colorectal cancer in a tertiary care hospital from November 2011 to November 2015. The study was approved by the institutional ethics committee. The diagnosis of colorectal cancer was based on colonoscopic biopsy examination and histological analysis of resected specimens.* S. japonicum* infection was detected by fecal or blood tests and/or histopathological examination of resected specimens. Fecal or blood tests to detect* S*.* japonicum* infection were performed in patients with suspected* S. japonicum* infection based on their clinical history or if they had a history of residing in areas where* S*.* japonicum *caused epidemics. All patients underwent radiological imaging such as ultrasound and contrast-enhanced computed tomography (CT) to determine the disease stage and to check for any complications of schistosomiasis, such as liver disease and ascites. In patients with underlying liver disease, Child–Pugh grading was performed. Patients with stage I–III cancer were included in this analysis. Seven patients received neoadjuvant therapy for preoperative rectal cancer of stage T3N0M0 or any T, N1-2M0, or T4 and/or locally unresectable M0 stage. The decision regarding the type of operation, that is, open or laparoscopic, was made by the patients after they were explained the benefits and risks of both approaches. Written informed consent was obtained from all patients before the operation. The standard surgical procedure was performed in all patients, depending on the location and extent of the disease. The surgeons in this study had more than five years of experience in laparoscopic colorectal surgery. Since schistosomiasis is known to cause peritoneal disease, multiple peritoneal biopsy samples were taken when suspicious peritoneal nodules were detected during the operation in order to rule out peritoneal metastases. Patients with multifocal tumors, those with acute intestinal obstruction or perforation due to colorectal cancer, pregnant women, patients with contraindications to laparoscopy, those who had undergone prior abdominal surgery, those who had a malignant disease in the 5 years prior to the study, those with Child–Pugh grade C, and those with massive ascites were excluded from the study (Figure 1).

Preoperative parameters included patient age, gender, body mass index (BMI), Child–Pugh classification grade, schistosomiasis-related concomitant diseases, the American Society of Anesthesiologists (ASA) score, and the volume of ascites. The operative parameters studied were operating time, operative blood loss, operative procedure, additional operations if any, conversion to open surgery, and the reason for conversion. Postoperative data included tumor size, depth of tumor invasion, disease stage, number of dissected lymph nodes, postoperative complications, time to first passage of flatus, days to first oral diet, duration of postoperative hospital stay, and schistosomiasis-related complications. The prothrombin time, serum albumin (ALB), and D-dimer levels on pre- and postoperative day 1 were compared.

Data were analyzed using the Statistical Package for the Social Sciences (SPSS) version 20.0 (SPSS Inc., Chicago, IL, USA). *P* < 0.05 was considered statistically significant. Continuous data were expressed as means ± standard deviation. Continuous variables were analyzed using the* t*-test, and qualitative variables were analyzed using the chi-square test.

The primary outcomes analyzed were short-term morbidity, mortality, tumor size, number of dissected lymph nodes, resection margin status, postoperative complications, duration of postoperative hospital stay, and schistosomiasis-related complications. The secondary outcomes studied were complication rates and quality of life measured at least up to 3 months after surgery.

## 3. Results

During the study period, 280 patients with schistosomiasis underwent colorectal surgery (87 patients, via the laparoscopic [LAC] approach, and 193 patients, via the open [OC] approach). The preoperative clinical data of these patients is summarized in Table 1. There was no statistically significant difference in the age, gender, BMI, ASA score, Child–Pugh classification grade, volume of ascites, and schistosomiasis-related concomitant disease between the groups.

The perioperative outcomes are summarized in Table 2. No statistically significant difference was observed with regard to the type of operative procedure, requirement of additional operation, and operative blood loss between the groups. One patient (1.1%) in the LAC group required conversion to open surgery owing to severe adhesions. The duration of operation was longer in the LAC group than in the OC group (180 ± 23 min versus 158 ± 19 min; *P* < 0.001). In the OC group, one patient developed splenic injury because of dense adhesions around the spleen. After 12 days of operation, the patient developed splenic abscess, which was percutaneously drained under ultrasound guidance.

**Figure 2:**
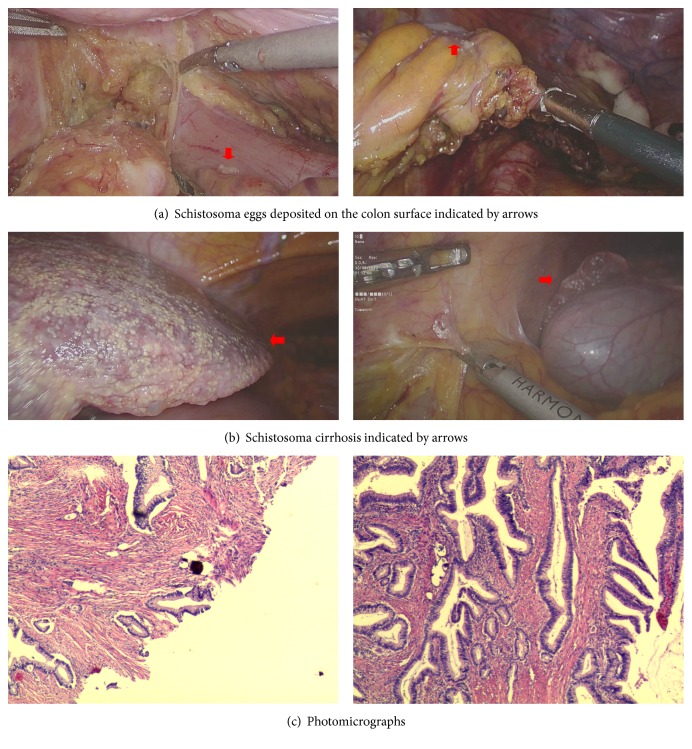
Intraoperative and histopathology images.

R0 resection was performed in all patients. Histological analysis showed eggs of* S. japonicum *in the resected specimens of all patients (Figure 2).  Table 3 shows the comparison of pathological characteristics such as tumor size, depth of invasion, number of lymph nodes harvested, and disease stage according to the Union for International Cancer Control (UICC) TNM stage.

Data regarding the perioperative outcomes is summarized in Tables 4 and 5. The days to first passage of flatus (2 ± 1 versus 4 ± 2; *P* < 0.001), liquid diet (5 ± 1 versus 6 ± 2; *P* < 0.001), and postoperative hospital stay (14 ± 4 versus 16 ± 7; *P* < 0.001) were shorter in the LAC group than in the OC group. The incidence of postoperative complications was lower in the LAC group than in the OC group (14 ± 4% versus 16 ± 7%; *P* < 0.001). Compared with patients in the OC group, those in the LAC group had significantly better postoperative albumin levels, which may be related to the minimally invasive surgery in these patients. No death occurred in either group.

## 4. Discussion

Several epidemiological studies have shown that there may be a causal relationship between schistosomiasis and colorectal cancer [8–11]. As schistosomiasis is a systemic disease, it also affects other vital organs such as the liver, leading to bleeding diathesis, which is likely to affect the surgical outcomes. Laparoscopic surgery has become the standard of care for treatment of colorectal cancer [12, 13]. However, whether laparoscopic colorectal surgery is safe and has better outcomes than those observed after open surgery in patients with schistosomiasis is not known.

In the present study, the mean operative blood loss was low and similar between the groups, despite the high incidence of bleeding diathesis in the study population. Moreover, the incidence of intraoperative or postoperative complications was similar between the LAC and OC groups. This suggests that laparoscopic surgery is at least as safe as open colorectal surgery in patients with schistosomiasis and its associated morbidities. The duration of surgery was found to be greater in the LAC group than in the OC group, which is consistent with previous findings [13–16]. However, the operative time is likely to reduce with increasing surgical experience [17, 18]. Moreover, the lymph node yield was similar in the two groups, which suggests that oncological clearance is not compromised with laparoscopic surgery. Fewer lymph nodes in some of the cases could be attributed to the use of neoadjuvant therapy.

The present study showed that laparoscopic surgery is superior to open surgery in terms of postoperative recovery, as reflected by the shorter time to first passage of flatus and stool, earlier intake of liquid diet, and shorter postoperative hospital stay. Similar findings were noted in previous larger studies [19–25]. However, the mean time for resumption of liquid diet was higher in this study, probably secondary to postoperative bowel wall edema, owing to the underlying hypoalbuminemia and portal hypertension.

The authors were concerned about the impact of schistosomiasis-related comorbidities on the perioperative outcomes. However, except for one patient in the OC group who developed splenic trauma due to dense perisplenic adhesions, no specific intraoperative difficulties were encountered. However, in the postoperative period, owing to the presence of liver disease, hypoalbuminemia (ALB < 25.0 g/L) was observed in 17 patients, which was corrected by infusion of human albumin. Variceal bleed was also observed in three patients in the OC group, which was treated using octreotide and endoscopic treatment. No increased incidence of these complications was observed in the LAC group. In order to evaluate the risk of venous thrombosis, the D-dimer values between the two groups were compared, and no significant difference was found. This result was similar to the results of Neudecker et al.'s study [26].

In conclusion, the present study, despite being retrospective in nature, suggests that laparoscopic treatment is safe and effective for colorectal cancer in patients with schistosomiasis japonica. However, only the short-term outcomes were analyzed in this study. Future studies with long-term follow-up are required to assess the oncological risks and benefits of laparoscopic colorectal surgery in patients with infections caused by* S. japonicum*.

## Figures and Tables

**Table 1:** Preoperative data.

	LAC (*n* = 87)	OC (*n* = 193)	*P*
Age (y)			0.367
Mean ± SD	68 ± 8	69 ± 8	
Range	(47–83)	(45–86)	
Gender			0.469
Male	46	111	
Female	41	82	
Body mass index (kg/m^2^)			0.457
Mean ± SD	26.5 ± 4.2	27.1 ± 4.5	
Range	(16.0–36.1)	(16.1–35.5)	
ASA score			
I	6	11	
II	65	142	
III	16	40	
IV	0	0	
Child–Pugh classification grade			0.406
A	69	161	
B	18	32	
Volume of ascites			0.367
None	79	168	
Minimal	8	25	
Schistosomiasis-related concomitant disease			
Liver cirrhosis	54	121	0.920
Esophageal and gastric varices	12	30	0.704
Splenomegaly	37	61	0.395
Perisplenitis	26	79	0.077
Leukopenia or thrombocytopenia	25	65	0.412
Colonic fibrosis	0	2	0.341

SD: standard deviation, LAC: laparoscopic, OC: open, and ASA: American Society of Anesthesiologists.

**Table 2:** Perioperative outcomes.

	LAC (*n* = 87)	OC (*n* = 193)	*P*
Operative time (min)			
Mean ± SD	180 ± 23	158 ± 19	<0.001
Range	(150–255)	(125–240)	
Operative blood loss (mL)			
Mean ± SD	95 ± 63	108 ± 85	0.196
Range	(10–300)	(20–600)	
Procedure			
Right hemicolectomy	18	58	
Left hemicolectomy	4	14	
Sigmoid resection	16	22	
Anterior resection	33	76	
Hartmann procedure	2	14	
Abdominoperineal resection	14	9	
Additional operation			
Oophorocystectomy	1	2	
Appendectomy	1	4	
Cholecystectomy	2	9	
Open conversion	1	—	

SD: standard deviation, LAC: laparoscopic, and OC: open.

**Table 3:** Pathological outcomes.

	LAC (*n* = 87)	OC (*n* = 193)	*P*
Tumor size (mm)			
Mean ± SD	43 ± 17	46 ± 20	0.197
Range	(10–60)	(10–70)	
Depth of invasion			
T1	9	11	
T2	12	27	
T3	52	110	
T4	14	45	
Number of lymph nodes harvested			
Mean ± SD	15 ± 8	16 ± 7	0.133
Range	(3–38)	(2–55)	
Pathological stage (TNM)			
1	17	32	
2	36	101	
3	34	60	

SD: standard deviation, LAC: laparoscopic, and OC: open.

**Table 4:** Perioperative outcomes.

	LAC (*n* = 87)	OC (*n* = 193)	*P*
Time to first flatus (d)			
Mean ± SD	2 ± 1	4 ± 2	<0.001
Range	(1–4)	(3–6)	
Time to liquid diet (day)			
Mean ± SD	5 ± 1	6 ± 2	<0.001
Range	(2–8)	(3–24)	
Postoperative hospital stay (d)			
Mean ± SD	14 ± 4	16 ± 7	<0.001
Range	(8–27)	(10–66)	
Postoperative complications	11	64	<0.001
Anastomotic leakage	2	10	0.270
Anastomotic bleeding	3	5	0.690
Abdominal bleeding	0	2	0.341
Wound infection	3	22	0.031
Abdominal infection	0	3	0.242
Pneumonia	2	9	0.346
Urethral infection	0	3	0.242
Ileus	1	4	0.589
Urinary disturbance	0	4	0.176
Chyle fistula	0	2	0.341
Schistosomiasis-related complications			
Upper gastrointestinal bleeding	0	3	0.242
Massive ascites	3	7	0.941
Severe hypoproteinemia	1	16	0.021

SD: standard deviation, LAC: laparoscopic, and OC: open.

**Table 5:** Comparison of perioperative prothrombin time (PT), serum albumin (ALB), and D-dimer levels between the groups.

	PT (s)		ALB (g/L)		D-dimer level (ng/mL)	
	Preoperative	Postoperative day 1	Preoperative	Postoperative day 1	Preoperative	Postoperative day 1
LAC (*n* = 87)	13.3 ± 0.7	14.5 ± 1.0	41.4 ± 4.6	33.2 ± 3.2	930 ± 1193	4600 ± 3319
OC (*n* = 193)	13.6 ± 0.8	14.8 ± 1.0	38.9 ± 4.7	31.0 ± 4.2	786 ± 862	3781 ± 3260
P (preoperative versus postoperative)	0.806		0.554		0.112	
